# Impact of pipelines on land use in rural areas in Turkey

**DOI:** 10.1186/s40064-016-2805-1

**Published:** 2016-07-22

**Authors:** Birol Alas

**Affiliations:** Architecture and Urban Planning Department, Vocational School, Okan University, Istanbul, Turkey

**Keywords:** Land use in Turkey, Easement rights, Pipelines, Cadastral data, Statistical analysis

## Abstract

This paper examines whether the parcelization of land through easement as a result of the construction of pipelines in rural areas in Turkey has any negative effects on productive land use. The current legislation in Turkey does not allow the division of land in most rural areas into parcels smaller than 5000 m^2^. Therefore, the smallest parcel that can be productively used was considered as 5000 m^2^. On the basis of the analysis of the data pertaining to the easement rights having two different widths and collected from three different regions having different parcel sizes, the research aims to find out the number of parcels with an area less than 5000 m^2^ (excluding the easement) that were created by the construction of pipelines and to investigate whether a significant number of areas less than 5000 m^2^ remain. This study also demonstrates a method that can be used in studies on this subject according to the various parcel sizes that were created by the allotment of parcels due to the easement of the construction of pipelines.

## Background

A pipeline is one of the most effective methods for transmitting energy sources like petroleum and gas (Ebrahimipoor et al. 2009; Iqbal and Satar 2006; Callan 2008; Hensel and Oelhaf 2004). Thus, it is the most preferred transmission method throughout the world (Yıldırım and Yomralıoğlu 2011). Two methods are used in the route planning of the pipeline. One of them is traditional method.

Traditional methods are used to determine routes for pipeline projects in Turkey (Yıldırım and Yomralıoğlu 2011). BOTAŞ (Naturel Gas Distribution Company of Turkey), which is responsible for the pipelines in Turkey uses the traditional methods in the routing process. Traditional methods of optimal routing in pipelines are mainly based on expensive and protracted methods. These methods are not precise, and the role of all effective parameters in pipeline routings cannot be considered easily. Most technical, economical and environmental concerns are not accounted for in design paths (Ebrahimipoor et al. 2009; Iqbal and Satar 2006).

Geographical Information Systems (GIS) technologies are another process used for determining the pipeline routing. Reducing construction costs, as well as potential environmental damages, and minimizing construction period of pipeline projects depends on appropriate route planning at the beginning of the process. With this aim, all factors which will have an impact on the route should be examined and analyzed in an integral manner. Depending on the distance between reserve and target destinations, frequent changes are observed in the surface (land use, topography, streams, etc.) and underground (soil, geology, etc.) characteristics, which result in a dense data set. Efficient management of this data and obtaining accurate results can be possible by using GIS technologies based on Raster Data Models (Yıldırım et al. 2008).

A study to compare the two methods showed that the route defined using raster network analysis techniques over the developed model reduces project cost by 23 %, pollutes the environment at a lower level, and is more appropriate from a sociological perspective. Moreover, current pipeline constructed by BOTAŞ using traditional method is seen to have less passage over agricultural fields (Yıldırım et al. 2008).

In Turkey, it is very common for the natural gas pipelines to run through rural areas. Thus, the need arises to establish the permanent easement rights concerning the parcels through which pipelines pass. The expropriation of permanent easement rights in Turkey does not change depending on whether the line is underground and aboveground. The permanent easements split parcels into fragments of various sizes. It is important to examine whether the negative impact of the pipelines on rural areas use is significant on the basis of the minimum area size requirements established by law in Turkey.

In this study it is researched whether the parcelization of land through easement rights as a result of the construction of pipelines in rural areas in Turkey has any significant negative effects on the productive land use of the parcels divided through easement rights. For this analysis, three underground pipelines that could be regarded as representative for Turkey were choosen. These pipelines used the traditional methods in the routing process has a length of 177 km in total. The area less than 5000 m^2^ was considered as the parcel area remaining out of use, because the current legislation in Turkey does not regulate the division of planted agricultural land and of land outside of the residential area that has not upper scale plan into parcels smaller than 5000 m^2^.

## Legal regulations in Turkey

There are various laws and regulations that regulate land use in Turkey. The pipelines pass through mostly the perimeter of the residential areas and the rural areas. Therefore legal regulations related to land divided in rural areas are important here. Regulations related to the division of parcels in rural areas were made by zoning ordinance based on “Zoning Law” No. 3194 and “Amendment of the Law on Soil Conservation and Land Use Law” No. 6537.

The Zoning Law regulates the conditions of the settlements and housing in these areas in accordance with the zoning plan as well as the requirements of protecting environment and health. Article 62 of the Unplanned Areas Zoning Regulation enacted on the basis of this law reads as follows (T.C. Resmi Gazete 1985a, b):

Article 62- (Amendend: RG-2/9/1999-23804) Each parcel that will be obtained after the allotment to be made outside of the residential area that has not upper scale plan cannot be smaller than 5000 m^2^. These parcels must have at least 25 m of frontage to the public road recorded in the land registry or land registry maps. The road cannot be created by using the method of abandoning parcels…

The aim of Amendment of the Law on Soil Conservation and Land Use Law is to lay down the principles and procedures for the conservation and development of soil, the classification of agricultural plots, to set the minimum sizes for agricultural tracts of land and for agricultural plots yielding sufficient income, to prevent their overdivision and to determine the ways conducive to the planned use of agricultural land.

Regulations for land in the agricultural area in “Amendment of the Law on Soil Conservation and Land Use No 6537” are as follows (T.C. Resmi Gazete 2014).

Article 4- … The minimum size of agricultural land can not be determined as less than 2 ha in absolute farmland, marginal farmland and special products farmland, less than 0.5 ha in planted farmland and less than 0.3 ha in farmland made greenhouse cultivation…

In addition, the definition of the minimum size of agricultural land made in Article 3. According to this, the division of land into smaller parcels more than 5000 m^2^ reduces productivity obtained from agricultural land.

Some other laws provide the provisions for registration and valuation of immovable property. Article 4 of the Turkish Expropriation Law reads as follows: “In place of the expropriation of immovable property, easement rights concerning certain parts, height, or depth of the immovable property or resources can be created through expropriation if they are appropriate for the relevant objectives.” Furthermore, Article 11 of the same Law stipulates that “in cases of the creation of easement rights through expropriation, the devaluation of the immovable property or resource arising from the act of expropriation should be stated clearly. This forms the basis for fixing the expropriation price” (T.C. Resmi Gazete 1983). In addition, Article 780 of the Turkish Civil Code reads as follows: “In order for easement rights to obtain, the registration of the property in the landbook is a precondition” (T.C. Resmi Gazete 2001).

In conclusion, the parcels which we examined owing to the construction of pipelines lie outside the residential areas and/or within agricultural land. In both of these rural areas, the minimum allotment condition is 5000 m^2^. The easement rights are recorded in deed and pipeline transit fees are paid in Turkey by establishing permanent easement rights.

## Methods

### Study design

In this study, three underground transmission pipelines constructed by BOTAŞ using the traditional methods in the routing process are examined. The parcels on which the three transmission lines run lie in the “rural areas” as delineated by Article 62 of the Unplanned Areas Zoning Regulation or Article 4 of Law No. 6537. This study rests on the assumption that the parcels with an area less than 5000 m^2^ as specified by these legal regulations be out-of-use land.

**Fig. 1:**
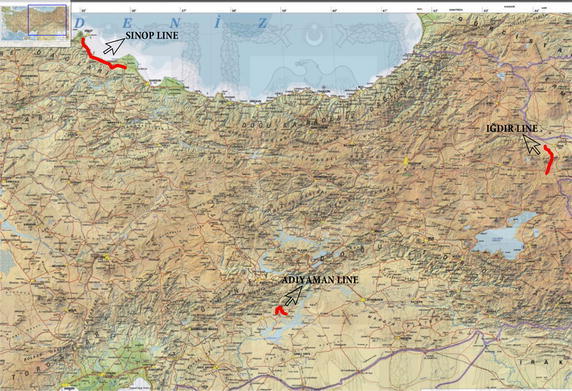
The pipelines in three different regions of Turkey (physical map of Turkey with scale 1/1,000,000)

**Fig. 2:**
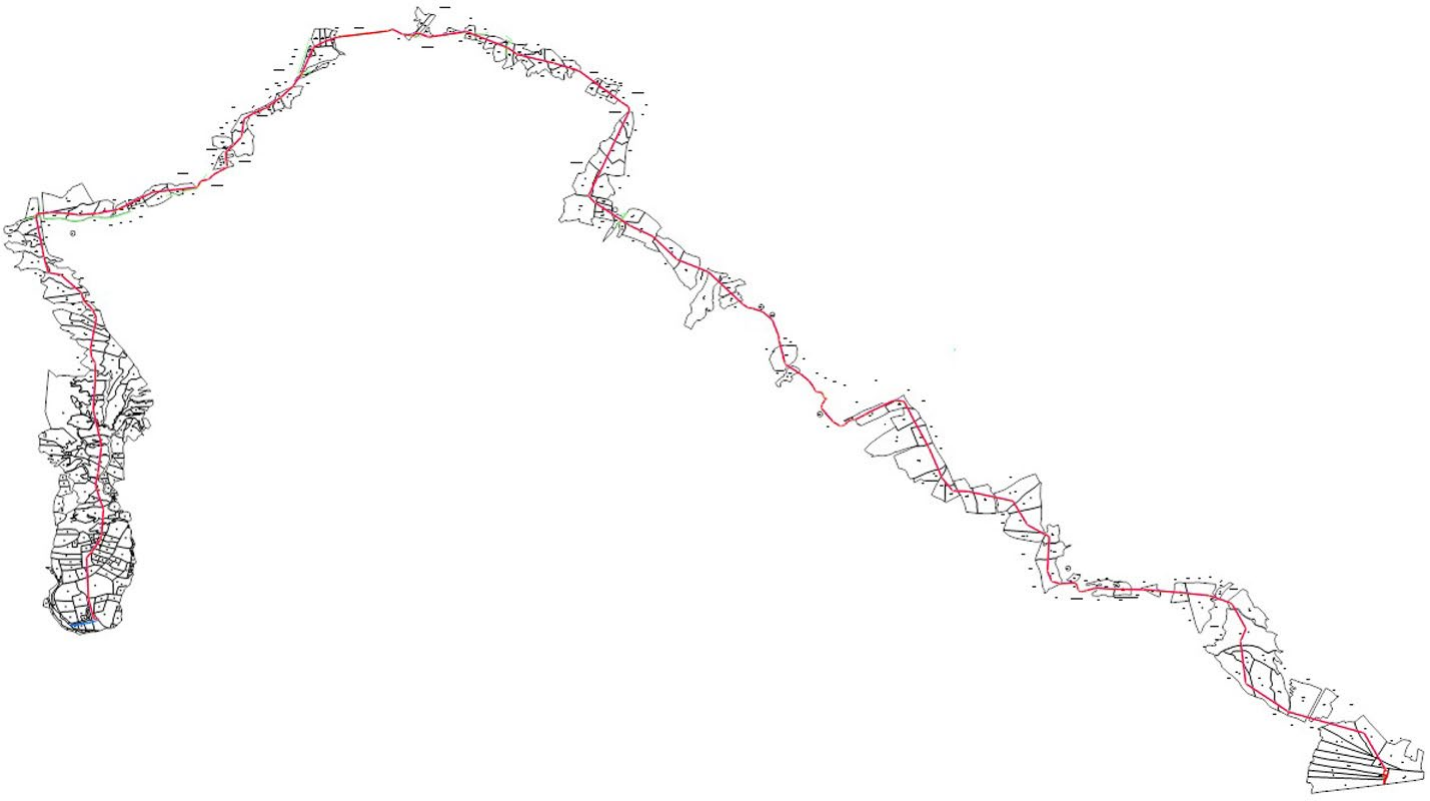
Adıyaman line (28.944 m long)

**Fig. 3:**
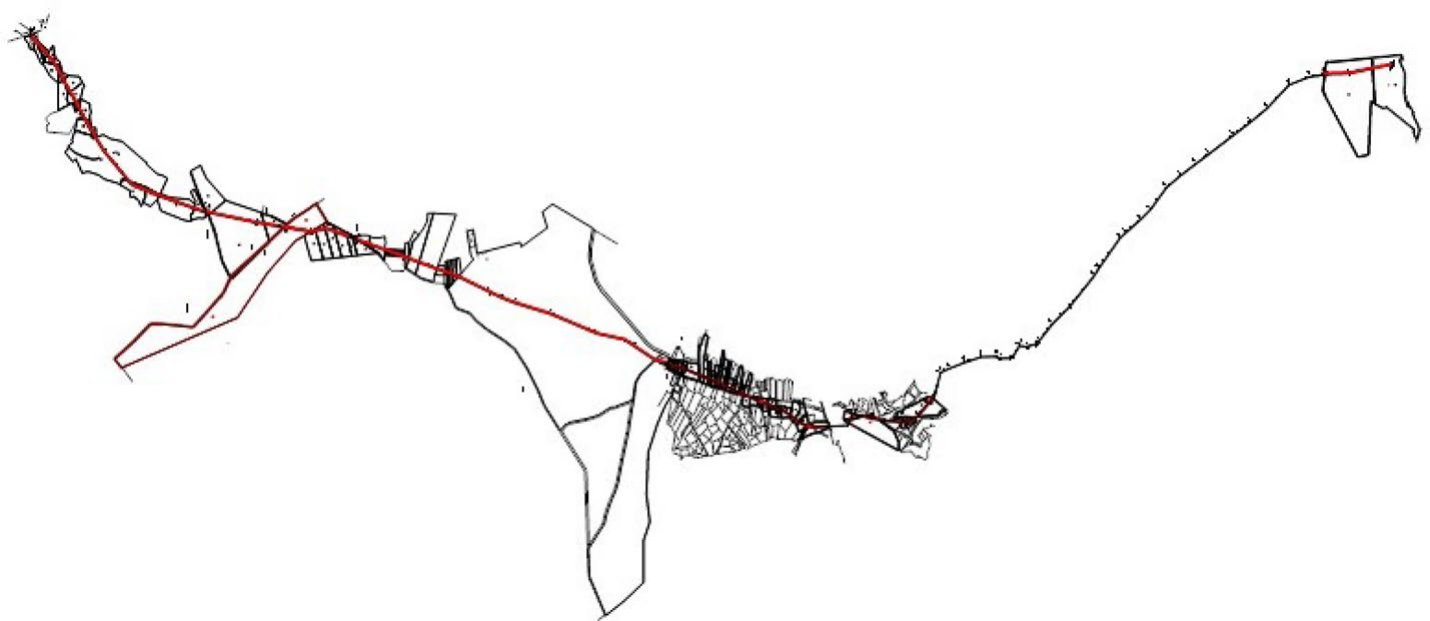
Iğdır line (37.840 m long)

**Fig. 4:**
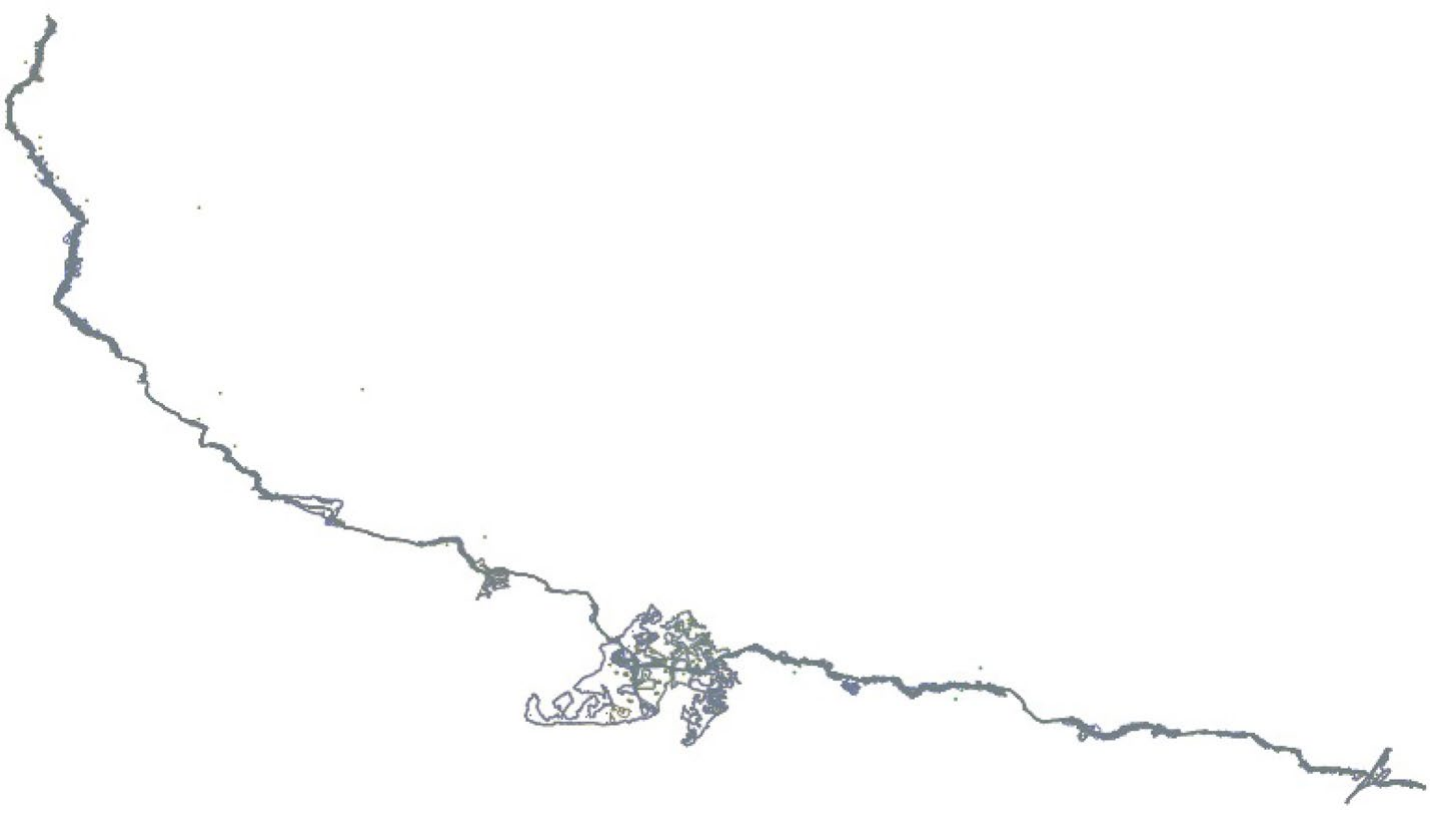
Sinop line (110.175 m long)

For this analysis, three pipelines that could be regarded as representative for Turkey were choosen (Fig. 1). Parcel sizes are different on all three lines and pipelines have two different easement width. The first pipeline investigated is the line in the province of Adıyaman (in the south of Turkey), running from Kahta to Menzil, which is 28.944 m long, and 6 m wide (permanent easement area wide) (Fig. 2). The second pipeline is the Iğdır line (in the east of Turkey), running from the district of Doğubayazıt (in the province of Ağrı) to Iğdır, which is 37.840 m long and 11 m wide (Fig. 3). The third pipeline is the Sinop line (in the north of Turkey running from the district of Bafra (in the province of Samsun) to Sinop, which 110.175 m in length and 11 m in width (Fig. 4). Line planning and land surveying of these lines was completed.

### Data collection

The data collected include the cadastral parcel data and the locations of the pipelines running through the parcels. The data on the route coordinates was obtained from the engineers who conducted the ground survey. The other relevant data was formed through the calculations of the size of the parcel fragments created by the easement rights on the basis of the examination of the land registers. The study rests upon the data generated by the measurements whose overall results are shown in Table 1. Data collected includes the following: City, county, village, map section, parcel number, deed area, remaining area on the left of the permanent easement on the departure direction, remaining area on the right of the permanent easement on the departure direction, permanent easement area (see Table 2).

**Table 1:** General condition of pipelines

Work name	Adıyaman natural gas transmission line	Iğdır natural gas transmission line	Sinop natural gas transmission line
Total kilometer	28.944	37.840	110.175
Pipe diameter	6 inc	10 inc	8 inc
Purpose	Natural gas transmission	Natural gas transmission	Natural gas transmission
Starting place	Adıyaman/Kahta/Çobanlı Village	Doğubeyazıt county	Samsun/Bafra county
Ending location	Adıyaman/Menzil/Durak village	Iğdır/Erhacı village	Sinop/Ordu village
Easement width	Right = 3 m, left = 3 m	Right = 7 m, left = 4 m	Right = 7 m left = 4 m
	Total = 6 m	Total = 11 m	Total = 11 m
Total number of some point	136	63	564
Total number of parcel	182	96	1331
Direction	East-South West	South-North	East-North West

**Table 2:** The data collected in 3 examined pipelines

City	County	Village	Map section	Parcel number	Deed area m^2^	Remaining on the left m^2^	Remaining on the right m^2^	Permanent easement area m^2^
Adıyaman	Kahta	Çobanlı	M41-A-17-C-3-B	120	44,800.00	23,536.62	19,402.41	1860.97
Adıyaman	Kahta	Çobanlı	M41-A-17-C-3-B	121	23,500.00	13,418.21	9767.63	314.16
⋮	⋮	⋮	⋮	⋮	⋮	⋮	⋮	⋮

### Data management

Firstly, the sum of the remaining area on the left and/or on the right less than 5000 m^2^ except permanent easement area for each parcel was found (the remaining area out of use). Secondly, the sum of the remaining area out of use and permanent easement area was found (the total affected area). Finally, the difference with the total affected area and the permanent easement area were examined by using statistical tests. The statistical analysis rests on the parcel sizes registered in the land book.

### Statistical analysis

In this study, statistical information was obtained by using the IBM SPSS Statistics 21 program.

### Descriptive statistics

First, descriptive statistics of the parcels, of the permanent easement areas, of the sum of permanent easement areas and remaining areas less than 5000 m^2^, of the remaining areas less than 5000 m^2^, of the deed area ratio of the permanent easement area, of the deed area ratio of the total affected area along the pipeline and of the difference between the total affected area and the permanent easement area were shown. Descriptive statistics calculated are shown in Table 3 for Adıyaman line, in Table 4 for Iğdır line and in Table 5 for Sinop line.

**Table 3:** Descriptive Statistics of Adıyaman Line

Description	Deed area	Permanent easement area	The sum of remaining areas less than 5000 m^2^ on the left and/or on the right	The total affected area: sum of permanent easement area and remaining area less than 5000 m^2^	The permanent easement area/deed area ratio	The total affected area/deed area ratio	Difference between the total affected area and the permanent easement area
N							
Valid	182	182	182	182	182	182	182
Missing	0	0	0	0	0	0	0
Mean	43,639.4966	900.3380	961.8804	1862.2190	.0952	.1774	961.8810
Std. error of mean	4056.06172	50.23994	126.27901	121.90267	.01630	.02160	126.27906
Median	28,686.7400	764.6650	.0000	1301,2600	.0200	.0400	.0000
Mode	16,800.00^a^	2.07^a^	.00	2.07^a^	.02	.02	.00
Std. deviation	54,719.26427	677.77388	1703.59698	1644.55694	.21984	.29140	1703.59765
Variance	2,994,197,881.820	459,377.429	2,902,242.666	2,704,567.534	.048	.085	2,902,244.961
Skewness	4.982	1.378	1.941	1.565	3.006	1.874	1.941
Std. error of Skewness	.180	.180	.180	.180	.180	.180	.180
Kurtosis	39.818	2.355	3.330	2.492	7.549	2.020	3.330
Std. error of Kurtosis	.358	.358	.358	.358	.358	.358	.358
Range	546,887.26	3770.48	8568.37	9103.83	1.00	1.00	8568.37
Minimum	172.74	2.07	.00	2.07	.00	.00	.00
Maximum	547,060.00	3772.55	8568.37	9105.90	1.00	1,00	8568.37
Sum	7,942,388.38	163,861.51	175,062.24	338,923.85	17.33	32,29	175,062.34
Percentiles							
25	13,512.5000	395.9525	.0000	790.3100	,0200	,0200	,0000
50	28,686.7400	764.6650	.0000	1301.2600	.0200	.0400	.0000
75	60,450.0000	1199.5700	1199.0150	2446.9525	.0400	.1400	1199.0150

^a^Multiple modes exist. The smallest value is shown

**Table 4:** Descriptive statistics of Iğdır line

Description	Deed area	Permanent easement area	The sum of remaining areas less than 5000 m^2^ on the left and/or on the right	The total affected area: sum of permanent easement area and remaining area less than 5000 m^2^	The permanent easement area/deed area ratio	The total affected area/deed area ratio	Difference between the total affected area and the permanent easement area
N							
Valid	96	96	96	96	96	96	96
Missing	0	0	0	0	0	0	0
Mean	283,228.3511	2825.0340	936.0905	3761.1246	.0386	.1010	936.0906
Std. error of mean	114,773.22166	596.11902	165.90731	594.57710	.00283	.01758	165.90732
Median	38,104.2000	1472.6700	.0000	2546.5000	.0300	.0400	.0000
Mode	11,000.00^a^	1644.83^a^	.00	1644.83^a^	.03	.03	.00
Std. deviation	1,124,543.31682	5840.74974	1625.55298	5825.64200	.02771	.17228	1625.55312
Variance	1,264,597,671,404.023	34,114,357.563	2,642,422.494	33,938,104.715	.001	.030	2,642,422.943
Skewness	7.358	7.380	1.541	6.987	.774	3.435	1.541
Std. error of Skewness	.246	.246	.246	.246	.246	.246	.246
Kurtosis	60.424	63.099	.802	58.961	−.007	13.212	.802
Std. error of Kurtosis	.488	.488	.488	.488	.488	.488	.488
Range	9,981,186.59	53,946.06	5052.55	53,844.88	.11	1.00	5052.55
Minimum	3493.00	10.99	.00	112.17	.00	.00	.00
Maximum	9,984,679.59	53,957.05	5052.55	53,957.05	.11	1.00	5052.55
Sum	27,189,921.71	271,203.26	89,864.69	361,067.96	3.71	9.70	89,864.70
Percentiles							
25	19,718.7500	830.5750	.0000	1214.2000	.0200	.0200	.0000
50	38,104.2000	1472.6700	.0000	2546.5000	.0300	.0400	.0000
75	95,837.5000	2828.5775	1171.6575	4813.7550	.0600	,0800	1171.6550

^a^Multiple modes exist. The smallest value is shown

**Table 5:** Descriptive statistics of Sinop line

Description	Deed area	Permanent easement area	The sum of remaining areas less than 5000 m^2^ on the left and/or on the right	The total affected area: sum of permanent easement area and remaining area less than 5000 m^2^	The permanent easement area/deed area ratio	The total affected area/Deed area ratio	Difference between the total affected area and the permanent easement area
N							
Valid	1331	1331	1331	1331	1331	1331	1331
Missing	0	0	0	0	0	0	0
Mean	37,163.8390	1284.1329	1560.0299	2844.1627	.2485	.6802	1560.0299
Std. error of mean	13,609.74174	447.09725	49.31948	448.29436	.00914	.01169	49.31948
Median	4084.8400	374.8300	912.5900	1751.0900	.1100	1.0000	912.5900
Mode	4.00	4.00	.00	4.00	1.00	1.00	.00
Std. deviation	496,522.47522	16,311.39209	1799.31632	16,355.06604	.33359	.42644	1799.31632
Variance	24,653,456,8400.116	266,061,511.979	3,237,539.202	267,488,185.049	.111	.182	3,237,539.202
Skewness	26.820	35,242	1.280	34.678	1.650	−.656	1.280
Std. error of Skewness	.067	.067	.067	.067	.067	.067	.067
Kurtosis	807.368	1268.773	1.417	1241.919	1.067	−1.482	1.417
Std. error of Kurtosis	.134	.134	.134	.134	.134	.134	.134
Range	15,887,349.74	588,851.51	9765.75	588,851.51	1.00	1.00	9765.75
Minimum	.16	.16	.00	.16	.00	.00	.00
Maximum	15,887,349.90	588,851.67	9765.75	588,851.67	1.00	1.00	9765.75
Sum	49,465,069.75	1,709,180.88	2,076,399.74	3,785,580.62	330.70	905.28	2,076,399.74
Percentiles							
25	1229.9000	120.3600	.0000	568.1500	.0500	.1500	.0000
50	4084.8400	374.8300	912.5900	1751.0900	.1100	1.0000	912.5900
75	10,350.0000	926.8200	2649.7000	3581.4300	.2300	1.0000	2649.7000

Some of the variables in Tables 3, 4, and 5, which are used for comparative purposes, are calculated in the following way.

The Permanent Easement Area/Deed Area Ratio: This ratio is calculated by dividing the permanent easement area size by the area size of the plot as registered in the title deed.

The Total Affected Area/Deed Area Ratio: This ratio is calculated by dividing the total affected area size by the area size of the plot as registered in the title deed.

### Examination of sample size

1. Determination of level of significance α = 0.05 (type I error)
2. Determination of test power β = 0.95 (type II error)
3. The smallest sample size n (total number of parcel) in the shortest pipeline is equal to 96
4. As a result of the taken as the standard deviation of the difference between the two areas of 1799 m^2^ (the biggest standard error between differences the total affected areas and the permanent easement areas of each lines), sample size was calculated using the formula given below (Sümbüloğlu and Sümbüloğlu 2005):

1 $$ n = \frac{{\left( {Z_{\alpha } + Z_{\beta } } \right)^{2} \times \sigma_{d}^{2} }}{{\delta^{2} }} $$

In the formula (1), Z_α_, Z_β_ the probability value of the standard normal distribution. $\sigma_{d}^{2}$ variance of a difference between the two measurements. δ^2^ meaningful magnitude for the review.

$$ n = \frac{{\left( {1.645 + 1.645} \right)^{2} \times 1799^{2} }}{{\delta^{2} }} = 96 \to \delta^{2} = \frac{{\left( {1.645 + 1.645} \right)^{2} \times 1799^{2} }}{96} \to \delta = 604 $$

In conclusion, with samples available, we could detect significant differences greater than 604 m^2^ at level of significance α = 0.05 and test power β = 0.95.

### Significance tests

#### Comparison of the pipelines in terms of parcel size

In this section was analyzed whether the parcel sizes of pipelines in three regions of Turkey are different using the test of analysis of variance.

### Hypothesis

#### **H**_**0**_

There is no significant difference between the pipelines in terms of the size of parcels.

#### **H**_**1**_

There is a significant difference between the pipelines in terms of the size of parcels.

#### Normality test results

The number of measurement was greater than 50 therefore Kolmogorov–Smirnov test was performed. Test was carried out for the “Deed Area” variables. Because 0.000 (Sig.) < 0.05 (for level of significance α = 0.05) the distribution of the all three groups did not have a normal distribution which are shown in Table 6.

**Table 6:** Normality test results of deed area of the lines

Tests of normality	Regions	Kolmogorov–Smirnov^a^			Shapiro–Wilk		
		Statistic	df	Sig.	Statistic	df	Sig.
Deed area	Adıyaman	.213	182	.000	.618	182	.000
	Igdır	.402	96	.000	.235	96	.000
	Sinop	.470	1331	.000	.039	1331	.000

^a^Lilliefors significance correction

#### Significance test results

There are three groups of “Deed Area” variables to compare. The tests that compare the three or more data sets were conducted since more than two sets were involved. The variables of the data sets are independent and include continuous numerical data. As a result of normality test, the non-parametric Kruskal–Wallis tests for several independent samples were performed.

A significant difference between the pipelines in terms of the size of parcels for level of significance α = 0.05 according to the test results which are shown in Table 7 (Sig. < α) was found. Another test was required to find that there is a difference between what groups. The Mann–Whitney U test was carried out to understand whether there are differences between pielines. As a result of the Mann–Whitney U test, the results of which are shown in Table 8 for the Adıyaman-Iğdır Lines, in Table 9 for the Adıyaman-Sinop lines and in Table 10 for the Sinop-Iğdır Lines, differences between the all pipelines (between the three groups) in terms of parcel size were found.

**Table 7:** Kruskal Wallis test results

Test statistics^a,b^	Deed area
Chi Square	362.406
df	2
Asymp. Sig.	.000

^a^Kruskal Wallis test

^b^Grouping variable: regions

**Table 8:** Adıyaman-Iğdır lines Mann–Whitney U test results

Test statistics^a^	Deed area
Mann–Whitney U	6431.000
Wilcoxon W	23,084.000
Z	−3.617
Asymp. Sig. (2-tailed)	.000

^a^Grouping Variable: regions

**Table 9:** Adıyaman-Sinop lines Mann–Whitney U test results

Test statistics^a^	Deed area
Mann–Whitney U	41,157.500
Wilcoxon W	927,603.500
Z	−14.464
Asymp. Sig. (2-tailed)	.000

^a^Grouping variable: regions

**Table 10:** Sinop-Iğdır lines Mann–Whitney U test results

Test statistics^a^	Deed area
Mann–Whitney U	10,922.000
Wilcoxon W	897,368.000
Z	−13.583
Asymp. Sig. (2-tailed)	.000

^a^Grouping variable: regions

#### Examination of the effect of the pipelines on land use in rural areas

In this study, it was mainly examined whether a significant difference between the total affected area and the permanent easement area. Our case is about the comparison of these two data sets, therefore the appropriate statistical tests that compare the two groups were carried out. The variables of the data sets are dependent on each other and include continuous numerical data. Therefore, this study was conducted with the Wilcoxon two related samples test (non parametric test) instead of paired samples t test (parametric test) since the assumptions of the latter test were not met (Akdağ and Sümbüloğlu 2010).

#### Hypothesis

##### **H**_**0**_

There are no significant differences between the total affected areas and permanent easement areas.

##### **H**_**1**_

The total affected areas are significantly greater than the permanent easement areas.

#### Normality test results

The number of measurement of three lines is greater than 50 therefore the Kolmogorov–Smirnov test was performed. Tests were carried out for the “Difference Between the Total Affected Area and the Permanent Easement Area” variable. For all three lines; because “Sig. (0.000) < α” is for level of significance α = 0.05 the distribution of the difference did not have a normal distribution (Table 11 for Adıyaman line; Table 12 for Iğdır line; Table 13 for Sinop line).

**Table 11:** Normality test results of Adıyaman line

Tests of normality	Kolmogorov–Smirnov^a^			Shapiro–Wilk		
	Statistic	Df	Sig.	Statistic	Df	Sig.
Difference between the total affected area and the permanent easement area	.316	182	.000	.639	182	.000

^a^Lilliefors significance correction

**Table 12:** Normality test results of Iğdır line

Tests of normality	Kolmogorov–Smirnov^a^			Shapiro–Wilk		
	Statistic	Df	Sig.	Statistic	Df	Sig.
Difference between the total affected area and the permanent easement area	.359	96	.000	.622	96	.000

^a^Lilliefors significance correction

**Table 13:** Normality test results of Sinop line

Tests of normality	Kolmogorov–Smirnov^a^			Shapiro–Wilk		
	Statistic	df	Sig.	Statistic	df	Sig.
Difference between the total affected area and the permanent easement area	.193	1331	.000	.832	1331	.000

^a^Lilliefors significance correction

#### Significance test results

Because of Normality Test Results, the non-parametric Wilcoxon paired sample test was performed. For all three lines; a significant difference between the total affected area and permanent easement area for level of significance α = 0.05 according to the test results which are shown in Table 14 (Sig. < α) for Adıyaman line, in Table 15 (Sig. < α) for Iğdır line and in Table 16 (Sig. < α) for Sinop line were found.

**Table 14:** Wilcoxon two related samples test results of Adıyaman line

Test statistics^a^	The total affected area: sum of permanent easement area and remaining area less than 5000 m^2^—permanent easement area
Z	−7.525^b^
Asymp. Sig. (2-tailed)	.000

^a^Wilcoxon signed ranks test

^b^Based on negative ranks

**Table 15:** Wilcoxon two related samples test results of Iğdır line

Test statistics^a^	The total affected area: sum of permanent easement area and remaining area less than 5000 m^2^—permanent easement area
Z	−5.232^b^
Asymp. Sig. (2-tailed)	.000

^a^Wilcoxon signed ranks test

^b^Based on negative ranks

**Table 16:** Wilcoxon two related samples test results of Sinop line

Test statistics^a^	The total affected area: sum of permanent easement area and remaining area less Than 5000 m^2^—permanent easement area
Z	−26.517^b^
Asymp. Sig. (2-tailed)	.000

^a^Wilcoxon signed ranks test

^b^Based on negative ranks

## Results

First of all, the descriptive statistics of the data sets have been obtained (see Tables 3, 4, 5). The average parcel size is 43.639 m^2^ for the Adıyaman line, 283.228 m^2^ for the Iğdır line and 37.163 m^2^ for the Sinop line. The average size of the parcels of land with less than 5000 m^2^ after the construction of the pipelines is 961 m^2^ for the Adıyaman, 936 m^2^ for the Iğdır and 1560 m^2^ for the Sinop lines. The average figure obtained by dividing the area size of the fragments of parcel with less than 5000 m^2^ by that of the plot as registered in the title deed is 2 % for the Adıyaman line, which runs through 182 parcels, whereas the same percentage is 0.2 % for the Iğdır line running through 96 parcels, and 4 % for the Sinop line running through 1331 parcels. Thus, the average percentage for all the three lines is 2.1 %. The calculations show that the ratio of the area size of the fragments of parcel with less than 5000 m^2^ to that of the plot as registered in the title deed rises as the number of parcels increases. Moreover, it is found that 75 of the 182 parcels (41 %) affected by the Adıyaman line now have fragments with an area less than 5000 m^2^, while the same figures are 36 out of the 96 parcels (38 %) for the Iğdır line, and 937 of the 1331 parcels (70 %) for the Sinop line.

Next, it was investigated whether the size of the parcels through which pipelines pass are different. A significant difference between the pipelines in terms of the size of parcels was found (see Tables 7, 8, 9, 10). Because the average size of the parcels affected by the construction of the pipelines is different for the three pipelines, the comparison of the pipelines in terms of parcel size could be also made.

Later, it was examined whether there is a significant difference between the total affected area and the permanent easement area using statistical related tests. According to the Wilcoxon two related samples test results, it was found a significant difference between the total affected area and permanent easement area for all three lines having two different permanent easement width and different parcel sizes.

It was concluded that the permanent easements formed as a result of the construction of the pipelines has created a significantly large number of land parcels with an area of less than 5000 m^2^ for the two different values of pipe diameters in all the three regions with different parcel sizes.

## Discussions

In the scope of some of the studies using GIS technologies, physical, environmental, political, social, economic and legal factors effective in the process from the planning to operation of pipelines are examined in an integral manner and implementations are carried out on the basis of “factor and weight” principle (Rylsky 2004; Saha et al. 2005). It is necessary to define the factors affecting route selection and the weight of these factors; to obtain the required data on the basis of these factors; and to organize this data in a database. Only by using GIS technologies, it is possible to take all of these steps in an integral manner and to make analyses for taking accurate decisions (Montemurro et al. 1998).

In the studies conducted to determine the weights required for optimal pipeline routing, the surface/underground conditions of the study area and the benefits expected from pipeline project and by making required examinations and analysis are taken into consideration. Weight can be changed over the model in case an alternative route need arises or when the model is to be implemented in any part of the country (Yıldırım et al. 2008).

When we look at in terms of expropriation, we see that parcel size are not taken into consideration in the Turkish Expropriation Law. The aim of the expropriation is only to acquire relating area, it does not take into consideration geometry of the surrounding parcels. Therefore these parcels stay unsuitable for different purposes (Uzun and Yomralıoğlu 2005).

Besides, in Turkey, land expropriation gives rise to some problems both for the state and landowners. Basically, the origin of problem is the determination of land price in order to obtain the real value. Significant number of expropriation implementations cause disagreement between the state and owners and these cases are brought to court. The lawsuits against the expropriation implementations in Turkey are started to be brought to the European Court of Human Rights (ECHR). These cases constitute more than 25 % of the cases against The State of Turkey (Yomralıoğlu et al. 2007).

In studies done on this subject it seems that the size of the parcel is not taken into account. It is significant to be that parcel size remaining out of use is one of the factors weighted with its importance in the planning of pipeline routes.

## Conclusion

This study investigated, whether the division into plots of different sizes of parcels through easement rights as a result of the construction of pipelines in rural areas in Turkey has any negative effects on the productive land usage. The current legislation in Turkey regulates the division of land in most rural areas into parcels at least until 5000 m^2^ due to the drawbacks in term of zoning and agriculture. In this study this was taken as a base and irrespective of the easement width and of the parcel size, the fragments of land with an area less than 5000 m^2^ did come into being in all the pipelines as a consequence of these construction projects.

It was determined that it is necessary to plan the construction of pipelines with due consideration of the possible impact on land use too. Thus, it is needed to include the variable of land use related to the parcel size to the list of the weighted factors in the planning of pipeline routes whether a traditional model or GIS-based technology be employed.

The method performed in this study can be used in studies on this subject according to the different parcel sizes that were created by allotment of the parcels due to easement of the construction of pipelines.
